# Total prevention: a history of schistosomiasis in Japan

**DOI:** 10.1017/mdh.2022.11

**Published:** 2022-04

**Authors:** Alexander R. Bay

**Affiliations:** History Department, Chapman University, Orange, CA 92866, USA

**Keywords:** Yamanashi, Schistosomiasis, Public health, Toilets, Human waste, Total war

## Abstract

In Japan, schistosomiasis was endemic in Yamanashi Prefecture and a few other hotspot areas where the Miya’iri snail lived. The parasite’s lifecycle relied on the intermediary Miya’iri snail as well as the human host. Parasite eggs passed into the agrarian environment through untreated night soil used as fertiliser or through the culture of open defecation in rural Japan. Manmade rice fields and irrigation ditches, night soil covered paddies and highly refined growing seasons put people in flooded rice paddies to intensively work the land in the spring and summer. The disease was equally dependent on human intervention in the natural world as it was on the natural world intervening in the human body. It is important to stress the role of both the environment and culture in disease causation. This study posits that we view the pre- and post-war national mobilisation to remake the environmental and reform the culture of the rural sector to align with public health mandates and notions of hygienic modernity as a case of total prevention.

A traveler approaching Kōfu in Yamanashi Prefecture by train can visually appreciate the expression *bonchi,* or low-lying area. Kōfu is situated at the confluence of three rivers: the Fuefuki, Kamanashi and Arakawa (Figure 1). Historically, the area was swampy due to the gentle slope of the land, where the town’s three rivers frequently flooded. This environment, with rice-paddies in flood-prone low-lying areas next to rivers, was superb habitat for the *Oncomelana nosophora* snail. It could live in only a few areas in Japan and would be of little historical concern to us if not for its role in the lifecycle of the parasite *Schistosoma japonicum.* ^1^ This trematode causes the disease. Flukes in an aquatic environment burrow into the skin of a human or animal host. They travel through the bloodstream and find a mate; once conjoined, they produce eggs. Most are passed with feces into the environment but some remain in the body and cause the classic symptoms of snail fever: anemia, malnutrition, underdevelopment, dysentery-like diarrhea, enlarged spleens and livers that develop into cirrhosis and abdominal dropsy.^2^ Once the eggs reach water, through open-defecation or in nightsoil used as fertiliser in rice paddies, they morph and find the snail. Having gestated in the snail, cercaria emerge in rice paddies, irrigation ditches or slow-moving streams, and find a new host and infect them. The parasitic flatworm is dependent on the snail as an intermediary host; it shares a habitat with the snail and these areas became famous for their ‘local disease’.

**Figure 1:**
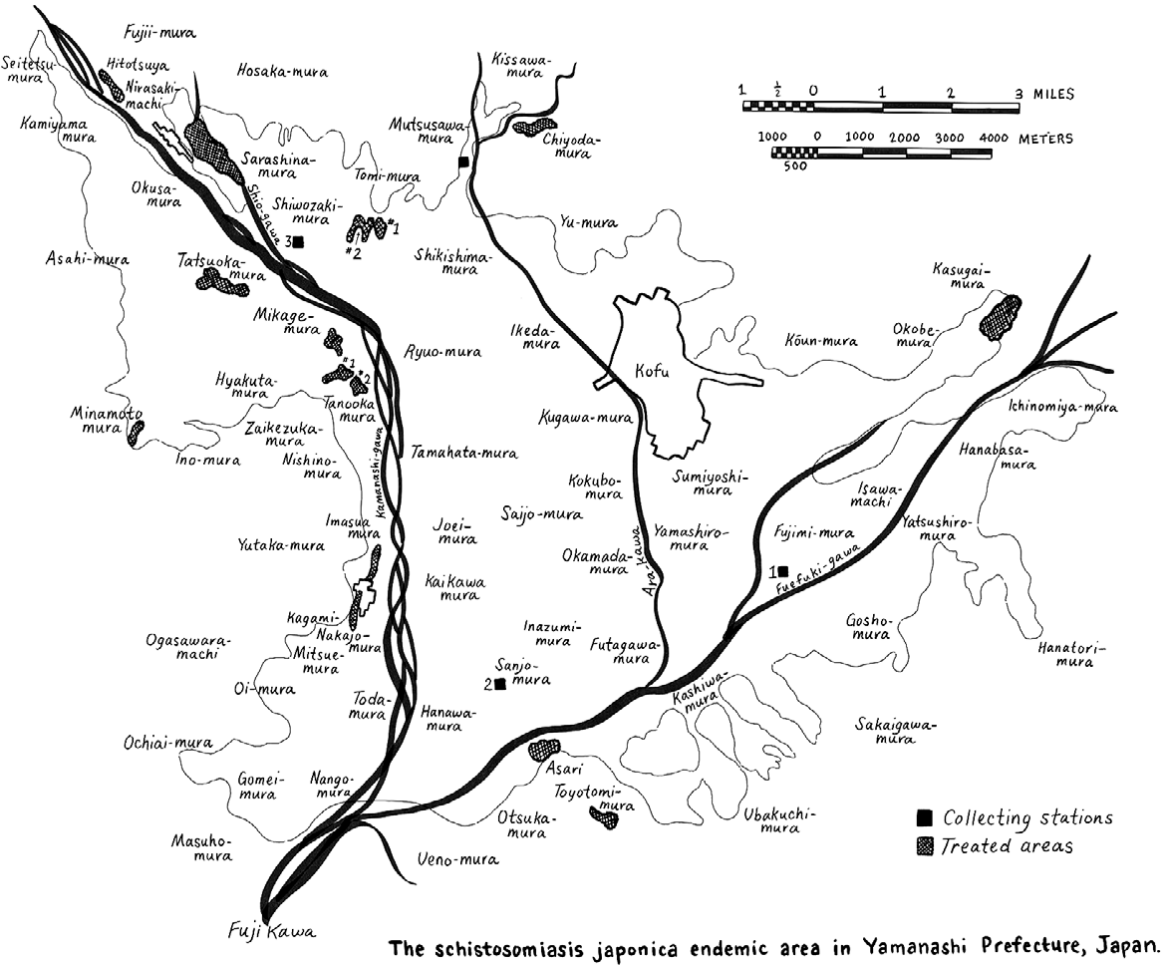
Adopted from Donald B. McMullen, S. Komiyama and T. Endo-Itabashi, ‘Observations on the Habitats, Ecology and Life Cycle of *Oncomelania Nosophora*, the Molluscan Intermediate Host of *Schistosoma Japonicum* in Japan,’ *The American Journal of Hygiene*, 54 (1951), 406.

The lifecycle of this parasite was embedded in the environment and the rhythms of agricultural life. A 1928 history of the Yamanashi ‘local disease’ wrote, ‘From the eggs in a patient’s feces, to snails, cercaria, to a person who then becomes infected, this route of disease propagation is *extremely circulative*, moving and developing from one to the next’.^3^ US Army Doctor Donald McMullen in 1951 noted that, ‘Rice culture and *Schistosoma japnonicum* seem to go hand in hand’.^4^ The consensus of experts was that the rural environment and agriculture created the opportunity for schistosomiasis infection. More recent critical literature on the environmental history of disease suggests that notions of a division between the human and the natural world need to be rethought. Human bodies and other organisms are a part of the surrounding environment and we cannot separate one from the other.^5^ Manmade rice fields and irrigation ditches, nightsoil covered paddies and highly refined growing seasons that put people in flooded rice paddies to intensively work in the land in the spring and summer suggest that this disease was equally dependent on human intervention in the natural world as it was on the natural world intervening in the human body. Due to the environmental nature of this disease, Dr McMullen argued that prevention was difficult because it involved ‘problems in engineering, agriculture, disposal or treatment of definitive hosts, and sanitation as well as the use of molluscicides. Anything less than a total effort is doomed to failure’.^6^

The story of schistosomiasis in Yamanashi Prefecture is a 100-year effort, from the 1880s to the 1980s, to rebuild the agricultural environment and engineer a farming culture in which the parasite could not propagate. Doctors imagined a ‘total war’ against parasites and snails. During the Second World War, Japan waged total war in Asia and the Pacific that required the deployment of all human and natural resources against the enemy.^7^ The economy, the social and political body, and culture had to be mobilised.^8^ Borrowing the motif of total war, I suggest we see the battle against the disease schistosomiasis as a case of ‘total prevention’. Doctors and public health officials used science to reveal the role of humans and snails in the lifecycle and biology of the parasite. After these early medical break-throughs, prevention campaigns enrolled local and regional politicians to back anti-snail or toilet-reform projects. Politicians then reached out to the central government for more funding. Education focussed on improving hygiene consciousness at the rural level. Farmers had to be taught about the ‘local disease’ and convinced to adopt new toilet and hygiene practices through prevention programmes. Children learned about the ‘local disease’ in school. Rural culture had to change. After the Second Worls War, the medical community worked with US Occupation doctors to further the total prevention effort. Local groups across Japan assembled to form a national anti-schistosomiasis association to further press the central government for funding. Mass media campaigns mobilised Kōfu city and Yamanashi Prefecture to support joint snail eradication and agricultural reform initiatives. Environmental reforms were couched in terms of the economic interests of farmers. Funds were directed at spreading molluscicide across snail habitat. Snails were not keen to cooperate, however. The natural world pushed back. Snails adapted, developed resistance to chemicals and snail colonies rebounded. Money was then provided to turn paddy fields into fruit orchards. Irrigation ditches were concreted over. Snail habitat was slowly destroyed. In short, the total prevention enterprise had to reform society, politics, the economy and the environment. All sectors of rural society including school children, teachers, agriculturalists, doctors, politicians and public servants were mobilised akin to total war to realise disease eradication.

Total prevention sheds light on how local initiatives intersected with larger national issues of empire, war and national rebirth in the post-war era. While roundworm and hookworm were far more prevalent, with infection rates reaching 70% of the rural population, the effects of schistosomiasis reverberated nationally and internationally. Endemic areas were famous for producing men who could not pass the army physical examination. The presence of this disease associated Japan with other third world, tropical disease hotspots such as the Nile River, famous for Bilharzia. Japanese doctors referred to their country as ‘a nation of parasites’.^9^ Nation making and empire building, as well as notions of hygienic modernity, required schistosomiasis prevention and eradication.^10^

## Environmental historiographies

As a tropical disease, schistosomiasis was seen through the lens of empire. John Farley in *Bilharzia: A History of Imperial Tropical Medicine* argues that the approach of Western medicine to prevention and treatment was always *imperial* in nature. The understandings of and solutions for schistosomiasis prevention were imposed by Western-trained doctors from abroad. Local inhabitants were excluded from the planning and running of these campaigns.^11^ Iijima Wataru notes that scientific knowledge about schistosomiasis in Japan, by Japanese doctors in this case, was constructed in the context of empire. He also shows the continuity of colonial medicine into the post-war era, as scientists with field experience in the colonies ‘were appointed to influential posts in national universities and research institutes’.^12^

In Japan, Farley argues that anti-schistosomiasis campaigns had negligible long-term effects; instead, the modernisation of agriculture was the biggest contributor to eradication in disease hotspots.^13^ Farley’s conclusion misses some fundamental aspects of schistosomiasis prevention during the twentieth century. E. G. Pike, in his handbook *Engineering against Schistosomiasis/ Bilharzia*, notes that in Japan, laying concrete irrigation ditches throughout endemic areas was expensive but the costs were justified by the benefit to agriculture.^14^ In terms of total prevention, disease eradication had to go hand-in-hand with agricultural reforms. Translating public health interests into larger societal ones such as the modernisation of agriculture ensured that this project succeeded in the long-term.^15^ Total prevention in Japan problematises Farley’s assertions: the campaign against schistosomiasis was one of enrolling local, regional and national groups to realise the goal of eradication.^16^

The historical and archaeological records are not complete enough to reveal when and where the Schistosoma parasite evolved to take advantage of snails and animal hosts. There are two main theories, however. The out-of-Africa thesis holds that early hominids moved together with the parasite from the ‘green Sahara’ into north Africa, the Middle East, and then into Asia. Conversely, a second school posits that the parasite first developed in India and then later traveled via animal migration back to Africa one to four million years ago to infect early hominids.^17^ Be that as it may, by the nineteenth century, the parasite and necessary intermediary host snails were embedded in many locales around the world, including a few rural hotspots in Japan. The complex nature of the fluke’s multi-staged life cycle was unknown. The parasite evolved to take advantage of human ignorance: incomplete knowledge about the parasite allowed it to propagate.^18^ A necessary part of total prevention was eliminating this ignorance.

The earliest writing about schistosomiasis comes from the Hiroshima area. In 1847, Fujii Kōchoku (1815–1895) wrote about a peculiar affliction in the Katayama area of Fukuyama domain. After the Meiji Restoration, public health officials in Fukuyama Prefecture began investigating the local disease because this area was notorious for producing men who were unable to pass the Army physical exam.^19^ The first scientific breakthrough came when Kasai Kenji (1868–1927) found parasite eggs in a Katayama resident’s stool in 1903. The next year, doctors discovered parasites in cat and human autopsies. Dr Katsurada Fujirō (1867–1946) termed this fluke *Schistosomum japonicum* because it was similar to the parasite that caused Bilharzia.^20^ Miya’iri Keinosuke (1865–1946) discovered that a small snail was the intermediary host in 1913: this made the lifecycle of the parasite clear and facilitated prevention campaigns.^21^

One could argue from this point that scientists and doctors ‘disciplined’ schistosomiasis by turning it into an object of knowledge and ‘disciplined’ Japanese farmers by colonising their bodies with new, modern hygiene practices.^22^ The stakes and spoils of the story go beyond body-colonising narratives, however. First, this is not a story about men and ideas that were applied to the environment but rather the story of how scientists working with parasites and snails in the field and agriculturalists working the land developed environmental solutions for an ancient and dreaded disease.^23^ Second, the story is not another example of how the nation and capitalism sacrificed Yamanashi farmers on the altar of empire or high-speed economic growth. In the end, prevention worked and it eliminated the suffering caused by schistosomiasis. I write within a field of *Toxic Archipelago*-type historiographies, yet my article is a success story.^24^ But one with a clear trade-off between a pathological, disease harboring environment and one sanitised of parasites as well as other flora and fauna.

General prevention focussed on one of three strategies: killing parasite eggs in nightsoil, eliminating snails from the agrarian environment or protecting against infection through the use of petroleum-based salves or protective clothing for people who worked in aquatic environments. Treatment was never the main focus of public health, partly because reinfection was a possibility,^25^ but also because disease prevention was a means to modernise the agrarian sector. Deworming would not accomplish this. Public health officials envisioned national and local campaigns that would reengineer not only environments but local society as well. Through lectures, slide shows, science exhibits, posters and pamphlets, farmers would learn about the parasite, its environment and best prevention practices.^26^

The Chinese took a different approach. As Miriam Gross has shown in *Farewell to the God of Plague*, the emphasis in China was on prevention through mass mobilisation. The Yangtze River was historically a schistosomiasis hotspot. In the 1960s, the Chinese Communist Party public health officials focussed on treatment over environmental reform, and during the Cultural Revolution, in the context of collectivised farming, the barefoot doctors and university professors sent to the countryside formed the critical mass for sustained action. These were the people who brought the science of treatment and fostered the enthusiasm necessary to address this problem. Despite some successes, China’s stress on treatment was less effective in the long run than the Japanese effort to reengineer the environment through concrete irrigation ditches and riparian projects. Whereas in Japan, the disease was eradicated in the 1970s, in China today, incidence rates are once again rising in historically endemic areas of schistosomiasis.^27^

The story in Yamanashi is about building anti-schistosomiasis networks, engineering the environment and changing local culture.^28^ During the twentieth century, doctors and public health officials connected the science in Imperial Universities to doctors in the clinic, revealed the relationship between disease and human waste, highlighted snail habitat in rice paddies, critiqued rural society, reformed the environment and connected disease prevention to the economy and to national strength. I call this total prevention. The main challenge, to borrow an expression from Bruno Latour, was getting this network to hold together.^29^ In 1931, Dr Saitō Minami (dates unknown) wondered if the three challenges of lack of funds, lack of knowledge at the local level and lack of ability to successfully take on such a large scale, natural enemy could be simultaneously tackled without ‘crapping out’ halfway through.^30^ His prediction was spot-on.

## Early medical break-throughs

In Kōfu and surrounding areas, farmers called any disease that caused distended abdomens (typical in parasite infection) ‘harappari’ (swollen belly). Katsurada Fujirō (1867–1946), professor at Okayama Medical School, found unidentifiable eggs in the stools of patients with ‘harappari’ in 1904. He soon thereafter found a new parasite through autopsy. Since the causal agent was similar to that of Bilharzia, Katsurada named it *Schistosomum haematobium japonicum.* ^31^ Parasitologists at this time knew the causal agent and had the eggs in the feces of the patients, but they did not know how the parasite entered the body. Fujinami Akira (1871–1934) and Nakamura Hachitarō (d. 1945) saw the need to disrupt the parasite’s lifecycle, arguing that the eggs in feces ‘must be destroyed’.^32^ They discussed the best way to sanitise nightsoil without destroying its value as fertiliser. ‘It would be ideal if someone could invent such a device’, they agreed, but also foresaw difficulty with social engineering: ‘It would be impossible to physically enforce a regime of defecation in every house’.^33^ The longue durée story of prevention included the building and institutionalising of new toilets that would sanitise human waste and new ‘orificial orders’, to borrow Warwick Anderson’s wording, that disciplined farmers into hygienic poopers.^34^

Miya’iri Keinosuke (1865–1946) of Kyushu Imperial University found the intermediate host of *S. japonicum*, the soon-to-be-called Miya’iri snail, in Saga Prefecture in 1913.^35^ This was a major breakthrough. Doctors now understood the parasite lifecycle and could formulate plans for intervention. Miya’iri imagined that political resources would have to be mustered to eradicate this disease. He argued, ‘In the least, an entire county or prefecture must move to achieve any results’.^36^ From this point, doctors began mobilising a wide array of allies into their networks for disease prevention.

Disrupting the lifecycle of the parasite, the basis for the first approach to disease prevention in the prewar era, required frank discussion about poop. Miya’iri Keinosuke talked at length about the problem of open defecation in the agrarian environment. The paths around rice paddies were littered with human feces, he said. Here, ‘people get the urge, and then they *do the deed* around paths. When it rains, the feces flow into irrigation ditches and out comes the parasite, which immediately finds a snail to infect’. To keep parasite-egg-laden feces out of fields and ditches, it would be imperative for farmers to change their toileting practices in the countryside by stopping the custom of open defecation. ‘This is the plan for keeping the parasite eggs out of aquatic environments. If this is done, Japanese schistosomiasis can be prevented’.^37^

Through social engineering of the agrarian classes’ hygienic culture, farmers were urged to use toilet facilities and properly store nightsoil for an extended period to allow fermentation and putrefaction to occur, since this would kill the eggs in fecal matter. Nagao Yoshitomo (1876–1958) and Katō Sen’ichi (1894–1981) called for open defecation to be outlawed. Maybe fines would help curb this predilection, they mused, but ‘It would be best to create new customs that would make farmers stop open defecation on their own’. ^38^ Education was crucial: ‘Teachers must create the custom, in elementary schools, of telling children to never leave the house in the morning without properly defecating in a toilet’.^39^

The disease could also be prevented if all the Miya’iri snails were eliminated, Nagao and Katō argued, but this step was also the hardest to achieve. They focussed instead on total prevention: science and nature were to be deployed against the parasite. They experimented with chemical applications of copper sulphate, calcium cyanamide and lime. Flamethrowers or steam guns were thought to be effective in killing snails along irrigation ditches and in paddy fields. They speculated that introducing a natural predator into the environment might also be an option since ducks, frogs, carp or firefly larva had been known to prey on snail populations. Nagao and Katō also discussed burying snails, in concert with riparian works along river banks, but they concluded that simply burying snails would not make an area inhospitable to the intermediate host at a later date. Lastly, farmers could stop the cercaria from infiltrating their bodies. This was not easy to accomplish, Nagao and Katō noted, because it would be difficult to make farmers wear protective leggings and the like. The main challenge to any of these was the opposition of the agrarian class to consistently practice these measures outside the purview of public health campaigns.^40^

Doctor Narabashi Heisaburō (dates unknown) advocated eliminating the Miya’iri snail as the surest method for disease prevention. Whatever point in the lifecycle they targeted, he argued, prevention had to resonate with the economic interests of farmers, because ‘the hosts of this parasite are the extremely conservative farming folk of the countryside’.^41^ Other experts, like Miyakawa Yoneji (1885–1959), thought anti-snail campaigns were simply not economically feasible. Miyakawa maintained that the cheapest way to achieve prevention was through the management of human waste. ‘Snails are spread between fields and paddies. To kill all of these is not possible. We cannot kill all the fish in the river. The best short-cut to prevention is killing the parasite eggs in nightsoil’.^42^ According to Miyakawa, Japan’s problem with parasites, which damaged its reputation as a civilised nation, was a national problem of managing human waste.^43^ ‘The management of our national sewage requires proper storage of nightsoil and the natural powers of putrefaction and fermentation that kills parasite eggs. This is the most economical method of prevention’.^44^ Regardless of method, the general consensus of schistosomiasis experts was that prevention had to align with economic and cultural prerogatives and priorities of the agrarian sector.

## Hygiene consciousness at the rural level

Yamanashi doctors and public health officials built the tracks of a prevention network between the First and Second World Wars. In medical publications, they debated the best means for prevention and argued the merits of eradicating snails in the wild versus killing parasite eggs in feces. Many doctors saw the challenges of environmental engineering as less daunting than the prospects of rural social engineering. In the field, early prevention efforts focussed on anti-snail campaigns. This prefectural initiative depended on the involvement of local workers. Starting in 1925, the Yamanashi Prefecture Hygiene Bureau would hire sixty to seventy people from the afflicted area, train and educate them, and then during the spring snail eradication campaign period, appoint a director to oversee the operation. The eradication crew would spread lime over 400–800 hectares of infected land.^45^ When the work was finished, the crew was dismissed. The trickle-down potential of these efforts did not lead to proactive local projects, however. As soon as the prefectural authorities left, their anti-snail or anti-schistosomiasis campaigns came to a halt.

Meanwhile, public health officials also carried out education campaigns. The earliest extant popular prevention book was *I am a Local-Disease Professor: A Talk on Japanese Schistosomiasis* published in 1917 for use in Kōfu.^46^ Here, it is important to note with a bit of historical irony that locals had to be educated that their ‘local disease’ even existed. The story starts out with two schoolboys, Dekobō and Chamekichi, trying to catch fish while wading in a stream. They are summarily pulled out by a man in a suit and top-hat who tells them that what they are doing is quite dangerous. He is a professor of the ‘local disease’ who has come to warn the people about this grave issue. He asks the kids if they had heard of ‘local diseases’ at school. Nope, they said. What about parasites? No? Okay, how about Miya’iri snails, ever see one? No??? Well then, no doubt, you have never heard of the parasite *S. japonicum*, right? I did not think so. The Professor in short order explains what a ‘local disease’ is, what a parasite is, and what *S. japonicum* is as well. He then asks them to imagine what would happen if, out of a hundred locals, sixty were afflicted with these parasites. What if a single village or even a single household carried it at this rate?

There would be few people able to work and the village or house would become poor. It would not be just the village or house that would be poor, it would be the nation of Japan that would be poor. On top of that, our countrymen would get sick, their bodies would waste away, strong soldiers would disappear and we would become a weak nation. Therefore, ‘local diseases’ are ‘Poor Country, Weak Army’ diseases. If this disease spreads, the country becomes poor and weak and we will not be able to fight wars against Germany or China.^47^

We must rid ourselves of this parasite, the Professor says, before proceeding to tell them about the worm’s life-cycle and how to prevent the disease. He does not elaborate on prevention measures beyond the need to grease up with petroleum-based salves before entering rice paddies to prevent parasites from burrowing into one’s legs.

Interestingly, the pictures tell a different story. We see the Professor and the boys finding snails, people and animals attacked by small *kappa*-like monsters (water sprites), and eggs attacking a snail. We see a dog defecating next to a rice field, farmers destroying snails they have collected, and one image contrasting sick and healthy men. One can only assume that the book was meant to be a prop for a lecture. The heavy lifting of prevention would have to have been discussed along with these slides.

From this text, we can infer that rural society had little understanding of public or private health issues related to schistosomiasis. The Home Ministry established the Health and Hygiene Investigation Committee in 1916 to address the kind of poor health and hygiene ideology in the rural sector that *I am a local disease professor* depicted. Miya’iri was a member of this committee. Under his direction, a team started in Yamanashi Prefecture, examined Yamashiro, Sumiyoshi and Asai villages for over a year, and checked the level of sanitation and parasite disease morbidity. By 1922, the Home Ministry had examined nine villages. It then turned over the investigation to local public health officials; by 1928, 134 villages had been investigated. In this manner, an organisation and system were set up to surveil hygiene throughout the country. Fecal tests were carried out from 1923 to 1929, as nearly three million villagers were tested for parasites and malaria. The highest incidence rate was found in Yamanashi, where a stunning 97% of the population tested positive for parasites. By 1927, Yamanashi Prefecture had passed guidelines for town and village hygiene, with special attention to eliminating typhoid fever and trachoma, administering antiparasitic treatments, reforming toilet practices, eradicating flies and mosquitoes and maintaining general hygiene. The prefecture dedicated special funds for toilet reform in schistosomiasis areas.^48^

This rural survey led to the 1931 Parasite Disease Prevention Law. In 1927, doctors petitioned the prefectural assembly, the governor, and the Home Ministry for funds. The Home Minister, Suzuki Kisaburō (1867–1940), responded with a letter expressing hope that by the next fiscal year, he could get national funds to cover half the costs of prevention campaigns since ‘this is a great matter of the health of the people’. The following year the governor of Yamanashi Prefecture sent a letter to the Home Minister requesting national funds to fight schistosomiasis, arguing that ‘it is not simply an issue of the health and hygiene of the nation, but it also greatly influences local industry and the ideology of the people’.^49^ The implication was clear: without national intervention in this public health crisis, rural people might succumb to the affliction of socialism. The Diet responded within 3 years, passing the Parasite Disease Prevention Law in 1931 to support the fight against intestinal worms in the rural sector.^50^

## Combatting rural apathy

In the field, however, doctors uniformly lamented the cultural level of farmers and their lack of concern for parasite diseases. The rural mindset had to be taken seriously to overcome inertia against prevention plans. The roots of rural apathy, according to Dr Koizumi Makoto (1882–1952), ran deep; farmers were characterised by ‘laziness, never ending tedium, the safety of superficial knowledge, willful personal interpretations, self-conceit, and apathy’. These ingrained traits ‘should not be ignored’.^51^ Koizumi’s 1929 book *Japan, Nation of Parasites* maintained that Japan was ‘Number One’ on the list of parasite countries. Social reform was necessary, since the entire population did little to alleviate the problem.^52^ In addition to a cultural upgrade, rural life needed improvement. ‘If the conditions of life for children and families, especially their environment, are not clearly improved, and if the people, not just school children, are not treated for parasites, it is a matter of fact that they will not avoid further parasite infections’.^53^ Koizumi also advocated for increased discipline throughout the countryside, with a special focus on medicine and nightsoil. Initial treatment for parasites was not a silver bullet. For drug regimens to be successful, surveillance and diligence were necessary.^54^ If a medical worker did not administer a full dosage across several days, the ‘wives who make frivolous objections based on superficial knowledge’, or ‘ignorant fathers who are overbearing with self-conceit’, would prevent proper treatment and hinder local and national public health campaigns.^55^

If the people had no sense of what hygiene was, well-laid plans for parasite disease prevention based on the spread of education and hygiene ideology would come to naught. Okada Ryō’ichi (dates unknown), in the conclusion of a two-volume publication on parasite prevention, argued that the results would be as effective as ‘chasing flies away from the dinner table’.^56^ Social reform was necessary and ‘open defecation must be outlawed to facilitate toilet reform’. In addition to hygiene campaigns, large-scale fecal tests and the administration of anthelmintics, it was necessary to ‘neuter nightsoil’, Okada said, since human waste was used as fertiliser.^57^ Addressing the shift in hygiene consciousness at the rural level, Okada called for a thorough effort.

The way to plan this popularization is through lectures, pamphlets, flyers, moving pictures, exhibitions to show parasite damage inside the body, routes of infection, how to store nightsoil for disinfection so that no nightsoil that has not been treated is used as fertilizer, how open defecation is an evil custom, the need to wash fruits and vegetables before eating, the need to drink only clean water, and the need to cook meat and fish at high temperatures to kill any parasites therein. The masses need to be taught from the bottom up.^58^

Part of Okada’s research included a fieldwork project that catalogued, and thus made legible, local thoughts and practices concerning parasite infection. The data revealed an array of beliefs about causation and treatment. Superstition and religious practice informed many approaches to gut worms. A monk could invoke the power of the Buddha or Buddhist Law to ‘curse’ away parasites. Preventative amulets could ward off infection as well. Acupuncture and moxa were thought to be effective. Crude drugs and over-the-counter formulas were also popular. *Kaya* seeds, *makuri* (the marine algae *Digenea simplex*), pomegranate, and mugwort were in line with Chinese medicine deworming prescriptions and these products had natural anthelmintic properties. In short, country folk could try one or all of the following: an over-the-counter drug like *makuri*, a preventative moxa treatment, or a parasite suppression incantation performed by a Buddhist monk.^59^

Part of the bottom-up anti-parasite campaign consisted of toilet technology change. Since the lifecycle of the disease was extremely environmental, eradication efforts focussed on killing eggs in nightsoil as well as killing snails in nature. Chemicals could have been added to latrines to kill eggs but this would have hurt the value of nightsoil as fertiliser. Yamanashi public health officials maintained that toilet and septic system reforms had to be easy:

The everyday disposal and storage of night soil must not be complicated or the average farmer will not put up with such measures. In short, it must be simple, without elaborate steps, and must maintain its value as fertilizer. The best method is to reform toilet and septic systems so that until the night soil is removed as fertilizer, the natural fermentation kills all the parasite eggs in the cesspool.^60^

Doctors saw the need for a technological system to render human waste harmless yet still viable as fertiliser. Naitō Kazuyuki (d. 1933) wrote that ‘we can see that a lack of parasitic diseases in foreign countries is due to the proper disposal of human waste…In other words, we can avoid spreading dangerous fecal matter if we reform toilets through building water-based flush toilets’. Since this was not feasible at the local level for financial reasons, the focus was instead on building the so-called Reform Toilets and manure vats for fermenting night soil long enough to kill the parasite eggs.^61^

**Figure 2:**
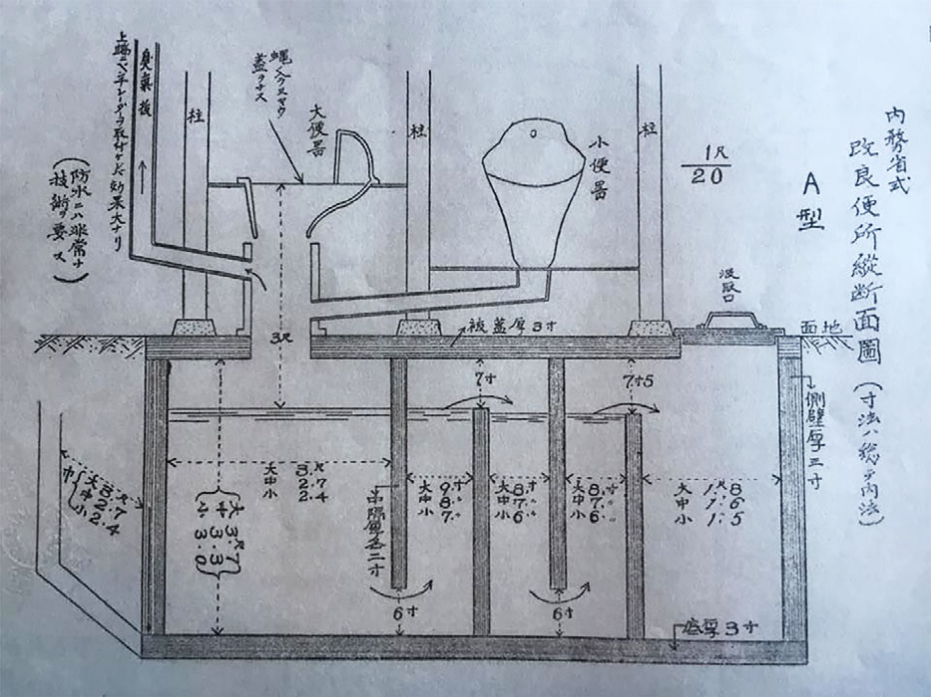
Tanaka Fuku’ichi, *Naimushō kairyō benjo sekōhō* (Shimane: Kamochō hōsha, 1932), 132.

Public health technocrats invented a new toilet to assure that prevention was sustainable. Nightsoil research revealed that if human waste was stored for over one-hundred days, anaerobic bacteria broke down organic matter by creating an oxygen-deprived environment that killed any and all disease-causing bacteria and parasite eggs. Dr. Takano Rokurō (1884–1960) developed the Home Ministry-Style Reform Toilet that was designed to kill microorganisms in raw sewage. (Figure 2) Takano wrote that, ‘Because Japan modernized its hygiene while ignoring nightsoil treatment and because it is the custom to use nightsoil as fertilizer, the main point of the Reform Toilet is to hygienically dispose of human waste’.^62^

In 1931, the government passed the *Parasite Disease Prevention Law* in part to encourage, through augmenting local coffers with national funds, the construction of more Reform Toilets.^63^ The law empowered local officials to carry out reform in the name of disease prevention, charging them with proactively addressing problems with human waste disposal. From the early Shōwa era, Reform Toilets were being built in northern Japan, the Kanto-region and western Japan. A single toilet/septic-tank cost between ¥20 and ¥40, depending on access to the sand necessary for concrete.^64^ Nationally, over 5 200 toilets had been constructed by 1931.^65^

Part of the Reform Toilet campaign was a pamphlet and manual for the construction of three- and five-chamber toilets. It laid out the rationale for and the pressing need for such a system. ‘The purpose of the Reform Toilet is to treat nightsoil in a sanitary manner and make it safe [for use as fertilizer] …Until we eliminate all the pathogens from human waste, it is only natural that fecal-oral route and parasite diseases spread’.^66^ Building toilets in the countryside and enlightening the rural population to proper sanitary practices were not equivalent. Kanie Yoshio (dates unknown), technical expert at the Nagayo City Hygiene Laboratory highlighted the need for everyday sanitary practices. ‘It is important to establish the custom of washing hands before eating. This is imperative: Hand washing not only protects against parasite diseases, but also prevents other fecal-oral route and infectious diseases’. Without bottom-up practices instilled at the local level, prevention would be impossible. Diligence was necessary. ‘There is no end to the embarrassment of being called the world’s preeminent parasite nation. We cannot be resigned to this unappealing stigma’.^67^ Doctors had difficulties, however, translating national problems with parasites into local agrarian concerns.

Okabe Hiroyoshi (1908–1974), an instructor of hygiene at Kyoto Imperial University, participated in a June 1937 field study follow-up to the Rural Health and Hygiene project. They found that in Kōfu, snail habitat was quite large, and the number of locals who understood schistosomiasis was rather small: schistosomiasis morbidity was 25.4%. Okabe recorded that,

The local villagers really have no sense concerning Miya’iri snails. When we are collecting them, the villagers look on with interest but they do not collect snails, even though they see us doing it. As we collect snails, many villagers pass us with indifference. We ask them about wearing long rubber boots. They respond, ‘We never wear those.’ We asked school kids about the snails and Japanese schistosomiasis, but they have absolutely no understanding. The villagers think that because the Prefecture runs their disinfection campaigns, that is enough. They know the name ‘local disease’ but they lack any real knowledge.^68^

Okabe hoped that the prefecture would pass some legislation to compel locals to eradicate snails and thus break the lifecycle of the parasite. Local officials and farmers did not participate in prevention without official oversight or coercion. Doctors worked to change local culture and practice but without the willing agency of farmers, they failed and the parasite lifecycle remained relatively unchanged.

Oral history recorded in Yamanashi also confirms a general lack of understanding of the ‘local disease’. In 1935, Kanemaru Tsuneno married into a farming family in Yatsuta-mura when she was 22 years old. She fell ill in 1938; her main symptoms were feeling lethargic and bloody stools but she recalled that at the time she had no idea what was wrong. Her in-laws did not help either. Only later did a relative tell her that she had the ‘local disease’ and that she needed to go to Dr Sugiura Saburō (1895–1977) in the neighboring village because he was the best at curing it. Sugiura treated her with thirty shots of Stibnal.^69^

## Wartime legacies during the US occupation

The nation mobilised for total war at the start of the second Sino–Japanese Conflict (1937–1945), and the government prioritised the health of soldiers and factory workers through proactive tuberculosis and beriberi prevention campaigns.^70^ As Japan moved aggressively into the Pacific, medical and state interest in tropical medicine also increased. The Dean of Ritsumeikan University, Matsui Moto’oki (1873–1947), penned the foreword for Yoshida Sadao’s *Tropical Parasite Diseases in Greater East Asia* in 1944. Matsui expressed his great expectations for the book to ‘contribute to the promotion of natural and technical science not only in Japan, but also for the larger East Asia Co-Prosperity Sphere’.^71^ Echoing Matsui’s sentiments as well as the spirit of the day, Yoshida (1878–1964) wrote that ‘The time has come for both the establishment of the Greater East Asia Co-Prosperity Sphere and East Asian science. As the leading power in East Asia, it goes without saying that we Japanese must be the leader of this’.^72^ Japan’s imperial ambitions were clothed in the rhetoric of Confucian humanism. Since Western imperialism was founded on the selfishness of individualism, Japan needed to rescue its Asian neighbors and help them build modern nations based on the idea of universal brotherhood. ‘With the outbreak of our Holy War’, Yoshida continued, ‘its purpose in terms of parasite disease research within the Great East Asia Co-prosperity Sphere is to better the welfare of the local populations. Our responsibility as leaders is great and heavy’.^73^ The medical and human sciences supported the war effort and many of these researchers became prominent in their fields after the Second World War.^74^

The rhetoric of empire changed radically after the Second World War; the message of public health and prevention, however, stayed the same. Yoshida Sadao, in the foreword for the inaugural issue of the *Japanese Journal of Parasitology*, invoked Emperor’s Hirohito’s speech announcing surrender,

Having extinguished the root of evil, the cause of our recent defeat, it is our fate to rebuild a peaceful nation. Young, old, men and women, all must rise up and exert themselves to the utmost…We will have to bear the unbearable and endure the unendurable with a strong and firm determination. We must awaken a will see it through to the end.^75^

Invoking the spirit of enduring defeat and sacrificing to rebuild Japan, Yoshida refocussed wartime rhetoric against parasites in the 1950s.

In the immediate post-war era, parasite disease incidence rates exploded. With food shortages, most nightsoil was recycled as fertiliser. Doctors tackled prevention through popular science publications. Iwata Masatoshi (1897–1997) wrote for a layman audience in *How we can protect against parasites.* The post-war boom in parasite diseases along with tuberculosis, Iwata warned, threatened the education of boys and girls. The first order of business was to spread correct knowledge concerning parasites: ‘I want the reader to know the main points of parasites, such as variety, symptoms, how to debug, test, and prevent infection’.^76^ Knowledge and power as well as science and the nation were part of the anti-parasite campaign to translate medical knowledge into everyday practice for total prevention.

At the same time that doctors worked to educate the masses, they were working with the Occupation forces to eradicate parasite diseases. After the Leyte campaigns during the Second World War, schistosomiasis was a concern for US military medicine. The occupation gave US Army doctors the chance to further their studies, test various molluscicides, and help the Japanese combat the disease. According to Donald McMullen and his Japanese counterparts, the Kōfu area was the most important. The lowing-lying region, ‘formed by the junction of three rivers’, was an ideal environment for the propagation of schistosomiasis. Studies in the late 1940s revealed incidence rates ranging from 38% to 65% in the Kōfu area. Public health workers and locals employed various attempts to kill off the parasite and intermediary host, including storing nightsoil in tanks, using calcium cyanamide (lime nitrogen), burning snail habitat, and making ‘radical changes in the irrigation system’, but, with incidence rates as such, there was no silver bullet.^77^ The approach aimed at eliminating the environments of the vector snail was problematic. McMullen and his team noted that parent colonies of snails lived in irrigation ditches and acted as bases for snails to move out into rice fields during the growing season. While molluscicides worked well to reduce numbers, unless repeated applications were made, the snail colonies would bounce back.^78^ Chemicals required more surveillance and this increased the participation from public health officials. The team concluded that, ‘Eradication of *Schistosomiasis japnonicum* in an area would necessitate a more or less continuous surveying and use of a molluscicide, until the snails were eliminated, or the human and reservoir hosts were no longer infected’.^79^

According to a 1951 US Army study, of the 3,055 people examined in Yamanashi Prefecture, 99.5% had some kind of parasite. Most had more than one infection. The average was 4.25 per person. Out of 3,055 people, there were 12,994 infections. Schistosomiasis made up 32% of the infections.^80^ While the US Army doctors prioritised molluscicides as quick fixes to be used in the field, since long-term social engineering was not feasible during combat operations, they noted that this approach was not best for civilian populations. Snail colonies would bounce back after chemical applications. Instead, a total prevention approach was necessary. McMullen argued that prevention ‘involves problems in engineering, agriculture, disposal or treatment of definitive hosts, and sanitation as well as the use of molluscicides. Anything less than a *total effort* is doomed to failure’.^81^

McMullen’s conclusion articulated well the aims of total prevention since snails developed resistance to chemicals and problematised the blanket use of molluscicide. Parasite prevention required a number of field-specific practices that eliminated snail habtat.

## Mass media strategies

In the post-war era, schistosomiasis experts worked to connect their local prevention networks with the larger national interests of rebuilding Japan. In 1948, the leaders of fifty-eight towns, cities and villages joined with doctors and local assemblymen to form the Yamanashi Local Disease Prevention/Eradication Union. The organisation’s job was to facilitate the spread of information, help carry out eradication, and co-fund prefectural projects.^82^ One of its members, Dr Sugiura Saburō, ran a hospital outside of Kōfu that specialised in treating the ‘local disease’. In the 1950s, Sugiura used Stibnal, the main schistosomicide at the time.^83^ The treatment was painful and required recovery time between shots. As an expert clinician and a local elite, Sugiura used his position to spread knowledge about the prevention and treatment of schistosomiasis. On Culture Day (3 November 1951), Sugiura and two school children read a script concerning ‘local disease’ prevention on the Kōfu Radio Broadcasting Station.

In the script, the doctor asks, ‘why they call it a ‘local disease’?’ The boy answers, ‘Because it is a disease limited to a particular area like Yamanashi or Shizuoka Prefectures’. The girl adds, ‘Yamanashi is said to have the world’s high incidence rate’. Sugiura asks if they learned about how parasites infect the body in school. The girl answers in the affirmative. ‘Through the skin, then to the heart, lungs or liver and end up living in the blood vessels of the intestine’. Sugiura asks how to avoid getting the disease. The girls responds, ‘By not going in water, I think’. Sugiura replies, ‘If you do not go in the water, then you should be able to avoid this disease. But what about the farmers who work in flooded rice paddies and irrigation ditches? If they cover their arms and legs with a salve containing benzoate they will be fine, but if they just wear boots or pants, cercariae can pass right through. The larger point is that to avoid this disease, parasite eggs should not be passed, that is, it is important to completely cure this disease’.

The boy asks if getting rid of all the Miya’iri snails would be the ticket. Sugiura says, ‘Exactly. The prefecture applies chemicals to kill them or it paves rivers with concrete so they cannot live there’. The girl asks whether firefly larva might help. The Doctor says that indeed there are numerous ‘natural predators’ such as crayfish that would help reduce the snail population. The boy mentions that nightsoil is dangerous and that this is a big problem for the farmers. Sugiura responds, ‘There has been and currently there is much research on some method to kill off all the parasite eggs in nightsoil, but if you let nightsoil ferment at a high temperature, the eggs will die’. The boy notes that if all the Miya’iri snails die, they can play in the water during the summer without fear. The girl adds that then the farmers would not have to worry either. Sugiura concludes with a speech:

You are correct. At any rate, because of this disease, we are despised by the people from other prefectures. If we do not do something to stamp this disease out, it will be to the great shame of the prefecture. Because of the damage to health, both strength and money is lost. This is a big deal. Every year we are using the nation’s money, ¥5,000,000, to kill snails. Therefore, we need cooperation and effort to quickly cast out this disease from our prefecture.^84^

Sugiura enrolled the history of this disease to shame farmers into action. The ‘local disease’ smeared the prefecture’s reputation and it was the duty of every Yamanashi resident to contribute to disease prevention and eradication not only for the prefecture, but also for the nation.

Despite Sugiura’s efforts, rural people still had little understanding of schistosomiasis. Sasamoto Kayo went to Yatsuta-mura in 1959 as a 20-year-old bride. After working in the rice paddies, she fell ill. When interviewed in 1979, she recalled having absolutely no understanding of this disease. ‘It was unbearable’, she cried. ‘No one in my husband’s family ever said, maybe it is the local disease’. A neighborhood auntie visited later and asked about her symptoms. ‘It must be the local disease’, she said, ‘You had better go and see the doctor’. Kayo got checked by the village doctor. He examined Kayo’s stool and said he had never seen so many eggs before. After showing her a slide under the microscope, he said, ‘You’re done for if you do not get treatment’. Kayo received approximately twenty-five shots of Stibnal.^85^

Yoneyama Tatsuo worked on ‘local disease’ prevention in Yamanashi Prefecture in the post-war era. He noted that around the same time, circa 1959, he received a letter from a bride who had come from outside Kōfu. She had no prior knowledge of the ‘local disease’. She fell ill and was diagnosed as having schistosomiasis; her mother-in-law however was rather indifferent about her plight so she found it difficult to get the treatment she needed.^86^ While this is an immediately recognisable ‘evil mother-in-law’ story, it reveals that the disease ecology of schistosomiasis was not only the bodies of the local agrarian population, but also the local farming society and culture. Indifference allowed the disease to propagate. As doctors had been saying for decades, total prevention would have to address rural apathy to eradicate this disease.

**Figure 3:**
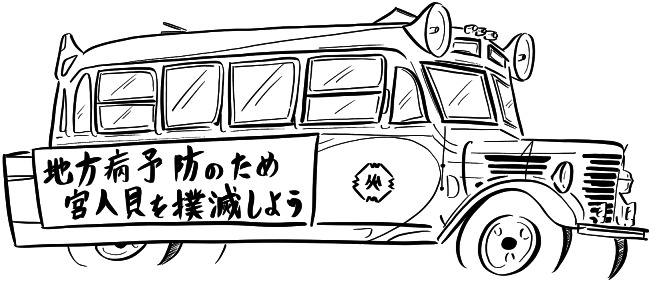
Adopted from *Chihōbyō to no tatakai: Chihōbyō ryūkō shūsoku he no ayumi*, ed. by Chihōbyō kinenshi henshū i’inkai (Kōfu: Yamanashi chihōbyō bokumetsu kyōryokukai, 2003), 61.

Part of the post-war total prevention campaign leveraged the influence of mass media to take the fight against the parasite and snails into the work spaces and classrooms of the people. In 1947, the city parked a ‘Parasite Train’ at Kōfu Station. It had a car dedicated to pathology research and one for fecal exams, as well as microscopes on display to show parasite eggs in slides and the like. In the 1950s, local governments ran ‘prevention cars’, a bus decorated with signs reading, ‘To prevent the local disease, let us eliminate snails’, and equipped with speakers to deliver the message to those within earshot (Figure 3). Yamanashi Prefecture Governor Amano Hisashi (1892–1968) participated in these events himself in 1955.^87^

The prefecture also ran consistent education campaigns during the 1950s and 1960s. It began in 1948 by distributing posters and leaflets, hosting lectures and putting on parasite exhibits. The prefecture included snail research in the middle school science curriculum. It also made fecal exams free. From 1954, the prefecture ran a month-long prevention campaign through exhibits at local health centers that included books on the parasite life-cycle, pictures, posters and schematics for reform toilets. Lectures were given and films were shown in endemic towns.^88^ In 1957 and 1958, the prefecture had an educational film and an illustrated book commissioned for spreading ‘local disease’ awareness. Elementary, middle and high schools ran schistosomiasis prevention essay contests and the winners’ entries were displayed at local health centers. These campaigns continued throughout the 1960s.^89^

In an attempt to keep children from playing in rivers and creeks during the summer, a typical activity as shown in *I am a local disease professor*, K-12 school principals were given the authority to sanction or forbid such recreation in 1949. This had limited effect since these activities happened outside the purview of the school principal. From the late 1950s, however, the prefecture began building pools for the elementary and middle schools to give kids somewhere to swim in the summer while keeping them out of parasite habitat.^90^

## Legislation and reform of agricultural practices

Environmental reform, through changes in agricultural patterns, was an important part of total prevention. In 1953, Yamanashi also set up the Local Disease Eradication Policy Promotion Committee that consisted of experienced medical personal to help decide the direction of prevention policy including the establishment of new parameters for effective irrigation ditch reform. The following year, public health officials amended the Parasite Disease Prevention Law to facilitate the flow of national funds to these local irrigation projects.^91^

The 1950s was the decade that pubic health officials tackled the nation’s human waste challenge. Robert Whiting in *Tokyo Junkie* wrote that the first thing he noticed about Japan in 1962 was the smell of raw sewage.^92^ Ladlers still removed most untreated effluent from cities to be recycled as agricultural fertiliser. Since raw sewage contained disease causing bacteria and parasite eggs, the technology of ladling had to change and a process for treatment needed to be institutionalised. In August 1952, the Japan Public Health Association meeting in Sapporo, in March 1953, the Japan Parasitology Association meeting in Fukuoka and then in May 1953, the Japan Hygiene Association meeting in Chiba all had a symposium and special lectures on parasite prevention and human waste management. On 9 May 1953, the Ministry of Health and Welfare Public Health Department established the Committee for Human Waste Management Policy. This focus made the 1950s the decade of dealing with human waste.^93^ As the economy developed after the war, ladlers and shit mongers often bought horses to pull larger carts, and later trucks to haul effluent out of urban areas, usually to river barges that shipped their payload to the hinterlands. Farmers, more often than not, did not compost this. In the late 1960s and early 1970s, municipalities built processing tanks to kill bacteria and parasite eggs in effluent before disposal. In the countryside, nightsoil collectors slowly phased out ladling and employed vacuum trucks to transfer human waste from household septic tanks to local processing plants.^94^

A 1953 roundtable discussion concerning parasites, including Komiya Yoshitaka (1900–1976) from the Parasite Division of the Hygiene Prevention Institute and others from Kitazato Institute, University of Tokyo, Gunma University and Toho University Medical School, argued that ‘the fundamental problem is human waste management’. ‘In the countryside’, they said, ‘in each village, someone needs to be charged with this type of work. If not, no matter how good the plan is, at this level of culture, it is impossible [to get the people to do it themselves]. The local administrative office must go through the trouble of overseeing this or all will be in vain’. They concluded that farmers needed to be disciplined to become hygienic poopers.^95^

**Figure 4:**
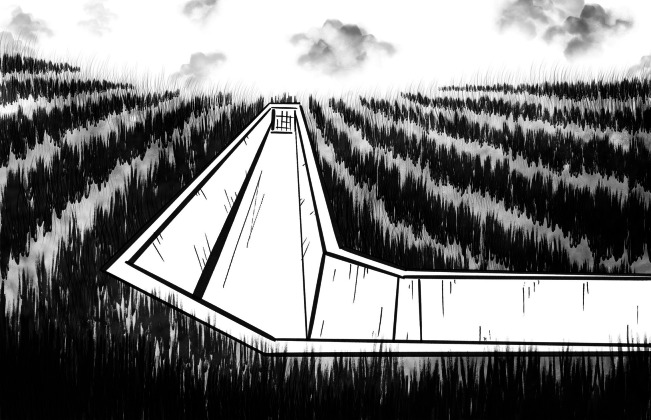
Adopted from Komiya Yoshitaka, ‘Miya’irigai (*Oncomelania nosophora*) no seisokuchi to sono satsumetsu no tame no konkuri-toka kōkyo no kanri jōkyō no chōsa,’ *Kiseichūgaku zasshi* 8:6 (1959), 88.

The need for surveillance extended to all aspects of the anti-schistosomiasis campaign. Yamanashi had been laying concrete in irrigation ditches to destroy Miya’iri snail habitat from the 1950s (Figure 4). Experts were also keen about killing snails with chemicals, but Miya’iri snails were extremely resilient. McMullen noted that snail colonies quickly bounced back after an application of molluscicide.^96^ Dr Sugiura, still in total war mode, argued that you could not let your guard down in the war against snails. ‘The enemy is a lower animal but its reproduction is vigorous. If we loosen eradication efforts it will bounce back stronger. Carelessness is the great enemy!’^97^ Snails resisted every attempt to kill them with fire, chemicals and concrete.^98^ Hosaka Yukio, a local Yamanashi public health official, noted in rhetrospect that molluscicides had limited value as a game changer in the battle against parasite and snails.^99^

Environmental engineering and concrete irrigation ditches were not silver bullets either, however. Constant upkeep was needed. Komiya Yoshitaka noted that ‘The purpose for laying concrete in irrigation ditches in Miya’iri snail habitat is to facilitate the steady flow of water, but another important reason is to eliminate the dirt and plants inside the ditch, thus starving the snails and making it impossible to lay eggs there’. Part of the management plan for the ditches would be to make sure that no vegetation in the surrounding area grew into the ditches. According to Komiya’s inspection, many were overgrown with vegetation even after concreting, thus providing snails with food and cover. This was bad. As he reported, ‘In Japan, the work of cementing such irrigation ditches is systematically promoted. But the present situation of their administration was found in bad condition in several areas’.^100^

Concrete irrigation ditches were part of disease prevention couched in terms of agrarian reform and progress. In 1958, the prefecture set up the Total Farming Management and Local Disease Policy and promotional flyers called for locals to ‘Eliminate the local disease and build a bright, healthy life! Reform agriculture and build an affluent life!!! First and foremost, we must eradicate the local disease from everyone’s district’. This particular print described how to use calcium cyanamide to kill snails.^101^ By combining agricultural reforms with prevention, this body carried out waste-water construction works, built infrastructure that changed the environment of arable land, and supported the switch from grain cultivation to fruit orchards.^102^ The prefecture along with local towns proactively turned mulberry trees (for silkworm cultivation) and paddies into peach and grape orchards.^103^ Oral history accounts attest to the prefecture’s support for turning problematic paddies into fruit tree farms and suggest that later understanding led to environmental engineering aimed at killing the parasite in the field.^104^ As a 65-year old woman lay dying from the effects of schistosomiasis, she told her husband to ‘eradicate this disease’. ‘I’m a goner’, she said, ‘a sacrificial victim’. ‘Do not let youngsters catch that disease’, she pleaded, ‘that area is covered with the local disease, fix it’. Her husband filled in the paddy lands to turn them into fruit orchards.^105^

In 1961, Yamanashi and other hotspot prefectures set up a national association, the All Japan Schistosomiasis Hot-spot Local Government Policy Committee to help promote the laying of concrete in irrigation ditches in endemic areas because the basic way to wipe out the snail vector was ‘by changing its environment’. This was not just disease prevention, but for the rural economy as well, Sasaki Takeshi argued. ‘Cementing ditches has also proved to be good for agricultural purposes’.^106^ With the spread of chemical fertiliser, the popularisation of farm machinery, and the increase of fruit tree orchards, the local land was changed to a drier environment less ideal for Miya’iri habitat. Due to these modifications and the continued use of molluscicides, incidence rates fell.^107^ By 1968, doctors reported that total prevention was working: ‘It is generally believed that a considerable reduction of human schistosomiasis in Japan has been brought about by the use of molluscicides, modification of snail habitat by cementing ditches, rapid development in agricultural method and industrialisation of the endemic areas’.^108^ In 1977, only three patients still remained in Yamanashi. The incidence rate had dropped from 5.47% in 1946 to 0.03% in 1977. From 1984, the disease was declared eradicated, although the prefecture was still dedicated to surveillance to make sure that the snail population did not return.^109^

## Conclusion

The overarching aim of prevention during the twentieth century was to disrupt the lifecycle of the parasite *S. japonicum.* Prewar public health campaigns envisioned a proactive agrarian population that would participate in toilet-reform or anti-snail practices. Education was a cornerstone of these campaigns because the local population had little to no knowledge about science, the lifecycle of parasites, or disease prevention. *I am a local disease professor* is a window into the rural society during the Taishō period that prevention campaigns hoped to reform. Okabe Hiroyuki’s 1937 report revealed agriculturists that had no interest in proactively addressing the cause of the local disease, and oral history accounts showed that the agrarian sector had little understanding of schistosomiasis. Dr Sugiura’s 1951 radio broadcast articulated a number of points that public health officials hoped were common sense. Again, interviews from former patients who contracted the local disease the late 1950s suggest that education was slow to diffuse to the popular understandings of health and hygiene.

In conclusion, how was ‘total prevention’ achieved? Doctors and public health technocrats had to transform and negotiate with the natural and human environment to make the network of prevention hold together.^110^ As parasitologists extended medical and scientific knowledge out into social practice, they had to modify their plans to accommodate the local agricultural ecosystem and the local agrarian society. Doctors wanted to remake both. They mobilised medical associations, schools, prefectural assemblies, governors, occupation forces and the central government. They also needed to enroll farmers, parasites, snails, irrigation ditches, septic systems and processing tanks into their network. Farmers and snails pushed back. Farmers remained ambivalent and snails developed resistance to molluscicides. Yet scientific knowledge slowly diffused to the agrarian population. Discipline and surveillance gradually brought farmers, snails and even concrete irrigation ditches in line with the aims of prevention. Alliances were made between public health officials and agriculture technocrats. Snail habitat was reworked to benefit both farming and disease prevention.

We should add that the economy did not simply develop and then yield social benefits. The economy and society were mutually constitutive. That is, schistosomiasis eradication was not simply the product of economic development but rather the result of a long-term project that made economics a central part of the prevention campaign. Farley wrote that all attempts to wipe out schistosomiasis in the tropics were imperial in nature, but that ironically it was the modernisation of agriculture that delivered the largest impact.^111^ In Japan, the two processes were combined. Disease prevention was part of a larger story of the imperialist tendencies of the state to wield science and technology to reorder society as well as the natural and built environment.^112^ Every time public health officials administered anti-parasite drugs, killed snails with chemicals, reformed irrigation ditches, concreted rivers, or improved rural infrastructure, they were doing so as the spokesmen for a larger network dedicated to total prevention – one that dated back to the 1920s.

The costs of total prevention included the destruction of local environments cherished by the local population. In 1972, 15-year-old middle school student Kitami Chieko, from Sado Island off the coast of Niigata Prefecture, wrote to the *Yomiuri shinbun* to complain about the loss of the natural environment due to the cementing over of the local river. The habitat for fireflies, purple lilies, dayflowers, rice fish and tadpoles had all been destroyed. The area where kids played in the river during the summer was also gone. Chieko understood that the cemented river helped agriculture, but she still did not like it.^113^ Another young reader from Yamanashi, the 13-year-old middle school student Nagata Kiyomi, responded to Chieko’s letter. Kiyomi wrote.

Ms. Kitami of Niigata, I also do not care for cement rivers but where we live we have Japanese schistosomiasis, a fearful ‘local disease.’ This parasite lives in the Miya’iri snail and this snail lives in stagnate water and mud. It cannot be found in steady-flowing streams. They made cement rivers call ‘local disease eradication canals’ to improve the flow…because of this, there are no more fireflies…but I think it is better than getting the ‘local disease.’ We still have people dying from it…There aren’t fireflies around the house but lotus and sunflowers bloom in the spring. We can hear the bush-warbler calling from the shrine forest. We hear frogs in the rice paddies. In fall, the crickets chirp to the point of being clamorous. What is most distasteful is that the main highway passes through our neighborhood.^114^

These middle-schoolers clearly understood the environmental losses related to development benefited local society. For the cost of summer swim spots or local firefly populations, they recognised the benefits of flood control and disease prevention. They also understood that the loss of habitat and their childhood interactions with nature was the price that local areas paid for national progress.

**Figure 5:**
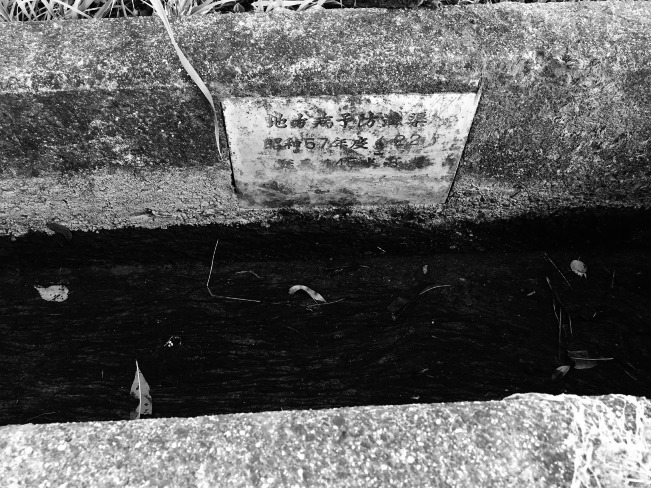
Photograph by author. The plate reads ‘Local disease prevention ditch, 1982’.

Fifty years later, total prevention in Kōfu is barely remembered. Tucked away in a neighborhood on the east side of the Kamanashi river is Dr Sugiura’s original clinic, now a museum. Across the river from the museum, in rice fields of the Kami’imasuwa area, one can occasionally find overgrown irrigation ditches that were laid during the eradication campaigns of the late Shōwa era (Figure 5). Today, if you asked what Kōfu was famous for, many would simply say ‘grapes’. Buried beneath concrete rivers, filled-in rice-paddies turned fruit orchards, and prefectural highways is the history of environmental and cultural change, aimed at preventing the ‘local disease’, dating back to the Meiji era.

## Footnotes

1 Katsurada Fujirō, ‘Yamanashi-kenka no chihōbyō ni tsuite’, *Okayama igakkai zasshi*, 173 (1904), 4; Donald McMullen, S. Komiyama, and T. Endo-Itabashi, ‘Observations on the habits, ecology and life cycle of *Oncomelania nosophora*, and the Molluscan intermediate host of *Schistosoma Japonicum* in Japan’, *The American Journal of Hygiene*, 54 (1951), 402; Kasai Kenji , ‘Bingo-kuni ni okeru iwayuru Katayamabyō chōsa hōkoku (zengō no zokukan)’, Tokyo igakkai zasshi, 18, 4 (1904), 30 ; Morishita Kaoru , Komiya Yoshitaka , and Matsubashi Hisakichi , Nihon ni okeru kiseichūgaku no kenkyū (Tokyo: Meguro Kiseichūkan, 1961), 105–10.

2

Fujinami Akira , Hiroshima-ken ni okeru chihōbyō Nihon jūketsukyūchūbyō zokushō Katayama-byō gaiyō (Fukuyama-shi: Kadota Insatsusho, 1926), 3.

3 Emphasis added. *Yamanashi-ken ni okeru Nihon jūketsukyūchūbyō gaiyō* (Kōfu, Yamanashi: Yamanashi-ken, 1928), 8.

4 Dr Donald McMullen was affiliated with the Walter Reed Army Institute of Research from 1945 to 1967. McMullen, *op. cit.* (note 1).

5 For the definitive study of the embedded nature of disease in the environment, see Linda Nash , Inescapable Ecologies: A History of Environment, Disease, and Knowledge (Berkeley: University of California Press, 2007)

6 Donald B. McMullen, ‘Schistosomiasis and Molluscacides’, *The American Journal of Tropical Medicine and Hygiene*, 1 (1952), 678.

7

Yamanouchi Yasushi , ‘Total War and Social Integration: A Methodological Introduction’, in Y. Yasushi , J. Victor Koschmann , and Ryū’ichi Narita (eds), Total War and ‘Modernization (Ithaca, NY: East Asia Program, 1998), 3.

8

Louise Young , Japan’s Total Empire: Manchuria and the Cultural of Wartime Imperialism (Berkeley: University of California Press, 1999), 13.

9 Koizumi Makoto, *Kiseichūkoku Nippon* Gakugei sōsho series 6 (Tokyo: Iwanami shoten, 1929), 3–4, 130.

10 For more on the concept of ‘hygienic modernity’, or how public health and hygiene was used as a measure of a country and a people’s progress in Asia, see Ruth Rogaski , Hygienic Modernity: Meanings of Health and Disease in Treaty-Port China (Berkeley: University of California Press, 2004), 1–21.

11

John Farley , Bilharzia: A History of Imperial Tropical Medicine (Cambridge: Cambridge University Press, 1991), 3–4.

12

Iijima Wataru , ‘“Farewell to the God of Plague”: Anti-Schistosoma japonicum Campaign in China and Japanese Colonial Medicine’, The Memoirs of the Toyo Bunko, 66 (2008), 59.

13 Farley, *Bilharzia*, *ibid.,* 300.

14 E.G. Pike, *Engineering against Schistosomiasis/Bilharzia: Guidelines towards Control of the Disease* (London: MacMillan Publishers, 1987), 166–67, 171.

15 Bruno Latour, *Aramis or the Love of Technology*, Catherine Porter, trans. (Cambridge, MA: Harvard University Press, 1996), 57, argues that to go from project to object, ‘you have to get a whole list of things interested *in the project.* Some of these actors and actuators, are docile, loyal, disciplined old servants. But other elements have to be recruited, seduced, modified, transformed, developed, brought on board. they *allow* or *forbid* other alliances. They require; they constrain, they provide. We have to tie them in recruit them, sign them up, bring them on board. I may as well say it: we have to *negotiate* with them’.

16 The local history of the anti-schistosomiasis campaign has been well-documented in the *Chihōbyō to no tatakai* (Battle against the local disease) series oft-cited below. It is, however, an uncritical narrative of the triumphant march forward of medical progress. See the English translation of its basic narrative along with numerous historical pictures in Kajihara Noriaki and Hirayama Kenji, ‘The War against a Regional Disease in Japan: A History of the Eradicaiton of Schistosomiasis japonica’, *Tropical Medicine and Health*, 39, 1 (2011), 3–44.

17

Robert Bergquist , Helmut Kloos , Aynalem Adugna , ‘Schistosomiasis: Paleopathological Perspectives and Historical Notes’, in B.G.M. Jamieson (ed.), Schistosoma: Biology, Pathology and Control (London: CRC Press, 2017), 10–2.

18 See the introduction to R.N. Proctor and L. Schiebinger (ed.), *Agnotology: The Making and Unmaking of Ignorance* (Stanford: Stanford University Press, 2008), 4. Robert Proctor writes that ignorance as a ‘native state’ describes a mentality that at least we moderns want to progress away from. Here, I am not making a value judgement but rather stating the fact that the parasite evolved without human knowledge of its details.

19 Akira, *op. cit.* (note 2).

20 Katsurada Fujirō, ‘Nihon jūketsu kyūchū no Saga-kenka ni okeru kankei ni tsuite’, *Okayama igakkai zasshi*, 175 (1904), 1.

21 Sueyasu Yoshio and Nakamoto Momosuke, ‘Nihon jūketsukyūchūbyō yobōhō zokuron I’, *Chūgai iji shinshi*, 1038 (1923), 752–3.

22 For the role of power in the production of knowledge, see Michel Foucault, *Discipline and Punish: The Birth of the Prison*, Alan Sheridan, trans. (New York: Vintage, 1995), 29; David Arnold, *Colonizing the Body: State Medicine and Epidemic Disease in Nineteenth-Century India* (Berkeley: University of California Press, 1993) is the paradigm setter for exploring the use of public health to control colonial subjects.

23 Richard White, *The Organic Machine: The Remaking of the Columbia River* (New York: Hill and Wang, 1996), 4, argues that people knew nature through working in it, not apart from it.

24 Brett L. Walker, *Toxic Archipelago: A History of Industrial Disease in Japan* (Seattle: University of Washington Press, 2011), 175, shows clearly that postwar Japanese intimately felt their nation through the bodily effects of industrial pollution disease such as mercury poisoning.

25 For more on reinfection rates, see Alexander R. Bay, ‘Disease, Environment, and Causation: Roundworm Prevention and Eradication in the twentieth-century Japan’, *Historia Scientiarum*, 27, 2 (2018), 150–73.

26 Fukutani Atsushi, ‘Nihon jūketsukyūchūbyō ni kan suru jikken hoi Nihon jūketsukyūchūbyō yobō bokumetsuhō tsuika’, *Kyoto igaku zasshi*, 24 (1927), 625. *Yamanashi-ken ni okeru Nihon jūketsukyūchūbyō gaiyō*, 24.

27 Miriam Gross, *Farewell to the God of Plague: Chairman Mao’s Campaign to Deworm China* (Berkeley: University of California Press, 2016).

28 Tsuji Moriyasu, ‘Kataki kara katayamabyō no bōatsu made’, in Miyairi Kenosuke kinenshi henshū i’inkai (ed.), *Jūketsu kyūchūshō to Miyairi Keinosuke: Miyairigai hakken kara 90 nen* (Fukuoka: Kyushu daigaku shuppankai, 2005), 29; Kusuribukuro Sho, ‘Yamanashi-ken no jūketsu kyūshūchū no bōatsu’, in Miyairi Kenosuke kinenshi henshū i’inkai (ed.) *Jūketsu kyūchūshō to Miyairi Keinosuke: Miyairigai hakken kara 90 nen* (Fukuoka: Kyushu daigaku shuppankai, 2005), 38.

29 Latour, *Aramis*, 213.

30 Saitō Minami, ‘Yamanashi-ken no Nihon jūketsu kyūchūbyō ha kakushite bokumetsu saretsutsu aru’, *Kōshū eisei*, 49 (1931), 657.

31 Fujirō, *op. cit.* (note 1), 1–44 (217–60). Fujirō, *op. cit.* (note 20), 1–16, 311–26.

32 Fujinami Akira and Nakamura Hachitarō, ‘Katayamabyō (Nihon jūketsu kyūchūbyō) no yobō’, *Chūgai iji shinpō*, 729 (1910), 1009–10.

33 Fujinami and Nakamura, ‘Katayamabyō (Nihon jūketsu kyūchūbyō) no yobō’, 1014–15.

34 Warwick Anderson, *Colonial Pathologies: American Tropical Medicine, Race, and Hygiene in the Philippines* (Durham, NC: Duke University Press, 2006), 106.

35 Miyairi Keinosuke, ‘Nihon jūketsukyūchū no nakama shukushu, tsuki, dōchūbyō no yobō’, *Tokyo iji shinshi*, 1839 (1913), 1–9 2121–28.

36 Miyairi, ‘Nihon jūketsukyūchū no nakama shukushu, tsuki, dōchūbyō no yobō’, 8.

37 Miyairi Keinosuke, *Kiseichūbyō yobobu chihōbyō yobō* (Tokyo: Naimushō eiseikyoku, 1921), 67–8.

38 Nagao Yoshitomo and Katō Sen’ichi, ‘Nihon jūketsukyūchūbyō no yobōhō ni tsuite’, *Nihon shōkakibyō gakkai zasshi*, 27, 5 (1918), 25–42, 291–308.

39
*Ibid.*, 27.

40
*Ibid.*, 27.

41 Narabashi Heisaburō, ‘Nihon jūketsukyūchūbyō no yobō’, *Chūgai iji shinshi*, 855 (1915), 1381–82.

42 Miyakawa Yoneji, ‘Kiseichūbyō II’, *Ikai jihō*, 1314 (1919), 2–3 (1458–59).

43 Miyakawa Yoneji, ‘Kiseichūbyō I’, *Ikai jihō*, 1313 (1919), 5.

44 Miyakawa Yoneji, ‘Kiseichūbyō III’, *Ikai jihō*, 1315 (1919), 4.

45 Saitō ‘Yamanashi-ken no Nihon jūketsu kyūchūbyō ha kakushite bokumetsu saretsutsu aru’, 656–58.

46 Yamanashi chihōbyō kenkyū-bu, *Washi ha chihōbyō hakase da: Nihon jūketsukyūchūshō no hanashi* (Tokyo: Seibidō, 1917), in Chihōbyō kinenshi henshū i’inkai (ed.), *Chihōbyō no tatakai: Chihōbyō ryūkō shūsoku he no ayumi* (Yamanashi: Chihōbyō bokumetsu kyōryokukai, 2003), 127–33.

47
*Washi ha chihōbyō hakase da,* 130.

48 Okada Ryō’ichi, *Kiseichū yobō ni kan suru chōsa hōkoku II* (Tokyo: Naimushō eiseikyoku, 1932), 1, 13–4, 156–57, 179–80.

49
*Yamanashi-ken ni okeru Nihon jūketsukyūchūbyō gaiyō*, 65.

50

Takano Rokurō , Kokuminbyō no yobō to bokumetsu (Tokyo: Ryūginsha, 1939), 320.

51 Koizumi Makoto, *Kiseichūkoku Nippon* Gakugei sōsho series 6 (Tokyo: Iwanami shoten, 1929), 118.

52
*Ibid.*, 12–3.

53
*Ibid.*, 120.

54
*Ibid.*, 122.

55
*Ibid.*, 129.

56 Okada, *Kiseichū yobō ni kan suru chōsa hōkoku II*, 737.

57
*Ibid.*, 743–44.

58
*Ibid.*, 747.

59 Okada, *Kiseichū yobō ni kan suru chōsa hōkoku I*, 614–39.

60
*Yamanashi-ken ni okeru Nihon jūketsukyūchūbyō gaiyō*, 7–13, 14.

61 Naitō Kazuyuki, ‘Kiseichūbyō no yobō’, *Kōshū eisei* 49 (1931), 636.

62 Naimushō eiseikyoku, *Shōkaki densenbyō oyobi kiseichūbyō bokumetsu jikken hōkoku* (Tokyo: Naimushō eiseikyoku, 1932), 129.

63 ‘Ikai zadankai: konshun no gakkai wo kataru (2)’, *Nihon no ikai*, 12, 15 (1931), 26.

64 ‘Takano-shiki benjo no fukyū: zenkoku de sude ni gosen amari’, *Nihon no ikai*, 21, 15 (1931), 27.

65 Miyazaki, ‘Kiseichūbyō yobōhō ni tsuite’, 643.

66 Naimushō eiseikyoku, *Shōkaki densenbyō oyobi kiseichūbyō bokumetsu jikken hōkoku*, 135.

67 Kanie Yoshio, *Kiseichū no hanashi: kujohō narabi yobōhō* (Nagoya: Nagoya-shi hoken-bu, 1933), in Kingendai shiryō hankōkai (ed.), Vol. 4, *Kindai toshi no eisei kankyō (Nagoya-hen) 9: shippei 4*, Kindai toshi kankyō kenkyū shiryō sōsho (Tokyo: Sanshinsha, 2013), 302, 291.

68 Okabe Hiroyoshi, ‘Yamanashi-ken Kōfu bonchi ni okeru Miya’irigai no seisoku jōtai narabi ni sono Nihon jūketsukyūchū kansenritsu ni oite’, *Kyūdai ihō*, 12, 1 (1938), 27.

69 Yamanashi Chihōbyō bokumetsu kyōryokukai, (ed.), *Chihōbyō to no tatakai (Taikensha no shōgen)* (Yamanashi: Heiwa Purinto-sha, 1979), 9–10. Stibnal was the main treatment for schistosomiasis from 1923.

70 For more on the history of tuberculosis in Japan, see William Johnston, *The Modern Epidemic: A History of Tuberculosis in Japan* (Cambridge, MA: Harvard East Asian Monographs, 1995), 252–293. For more on the history of beriberi, see Alexander R Bay , Beriberi in Modern Japan: The Making of a National Disease (Rochester, NY: University of Rochester Press, 2012), 128–151.

71 Yoshida Sadao, *Daitōa nettaiken no kiseichūbyō* shizen kagaku sensho 205 (Tokyo: Zokubundō, 1944), xii.

72
*Ibid.*, 2.

73
*Ibid.*, 21.

74 For more on the human sciences during wartime Japan, see Miriam Kingsberg Kadia, *Into the Field: Human Scientists of Transwar Japan* (Stanford: Stanford University Press, 2020).

75 Yoshida Sadao, ‘*Kiseichūgaku zasshi* no hankō wo shuku shite’, *Kiseichūgaku zasshi*, 1, 1 (1951), 1–4.

76 Iwata Masatoshi, *Kiseichū wo dōshite fuseguka* (Osaka: Tōyōzusho, 1949), 3.

77 Donald McMullen, S. Komiyama, and T. Endo-Itabashi, ‘Observations on the habits, ecology and life cycle of *Oncomelania nosophora*, and the Molluscan intermediate host of *Schistosoma Japonicum* in Japan’, *The American Journal of Hygiene* 54 (1951), 404–5.

78
*Ibid.*, 412–4.

79 Donald McMullen, S. Komiyama, and N. Ishi *et al*., ‘The Use of Molluscacides in the Control of Oncomelania nosophora, an intermediate host of Schistosoma Japonicum’, *The American Journal of Tropical Medicine*, 51, 5 (1951), 604.

80 G.W. Hunter III, L.S. Ritchie, and E.H. Kaufman*et al*., ‘Parasitological Studies in the Far East. IV An Epidemiologic Survey in Yamanashi Prefecture, Honshu, Japan’, *Japanese Medical Journal* 4, 2 (1951), 115.

81 McMullen, ‘Schistosomiasis and Molluscacides’, 678.

82
*Chihōbyō no tatakai: Chihōbyō ryūkō shūsoku he no ayumi*, 22–23.

83 Sugiura Saburō, ‘Nihon jūketsukyūchūbyō kagaku chiryō ni tsuite’, *Dai 20 kai sōkai gaikyō*, (1951), 29.

84 Sugiura Saburō, ‘Chihōbyō no yobō ni tsuite’, *Yamanashi kyōiku geppō*, 43 (1952), 21–3.

85
*Chihōbyō to no tatakai*, 3–6.

86
*Ibid.*, 8.

87
*Ibid.*, 15, 26, 61.

88 Health centers or *hokenjo* were local institutions set up as part of the Second World War total mobilization/total war campaign to maintain the health of the population for war support. Christopher Aldous and Suzuki Akihito, *Reforming Public Health in Occupied Japan, 1945–52: Alien Prescriptions?* (London: Routledge, 2012), 53–4.

89
*Chihōbyō to no tatakai*, 15, 25–6.

90
*Ibid.*, 25, 27.

91 Chihōbyō kinenshi henshū i’inkai (ed.), *Chihōbyō no tatakai: Chihōbyō ryūkō shūsoku he no ayumi* (Yamanashi: Chihōbyō bokumetsu kyōryokukai, 2003), 16, 22–3.

92

Robert Whiting , *Tokyo Junkie: 60* Years of Bright Light and Back Alleys…and Baseball (New York: Stone Bridge Press, 2021), 20.

93 Kodama Takeshi, ‘Kiseichū yobō to funnyō shori’, *Kōshū eisei* 14, 1 (1953), 3–7.

94 Alexander R Bay, ‘Disciplining Shit’, *Japan Forum* 31, 4 (2019), 556–82.

95 ‘Kiseichū ni kan suru mondai’, *Kōshū eisei* 14, 1 (1953), 14–24.

96 Donald B. McMullen, ‘Schistosomiasis and Molluscacides’, *The American Journal of Tropical Medicine and Hygiene* 1 (1952), 678.

97 Sugiura Saburō, ‘Nihon jūketsu kyūchūbyō’, *Nihon ishikai zasshi* 37, 9 (1957), 556.

98 Komiya Yoshitaka, ‘Nihon jūketsukyūchūshō no yobō’, in M. Kaoru, K. Yoshitaka, and M. Hisakichi (ed.), *Nihon ni okeru kiseichūgaku no kenkyū* (Tokyo: Meguro Kiseichūkan, 1961), 119. Tsutsumi Hiroshi, Hirata Mizuki, Takamori Kuni’aki, Chō Hiroyuki, Sasada Katsuyoshi, and Yoneda Yukata, ‘Chikugogawa ryūiki ni okeru miyairigai bokumetsu ni tsuite: Nihon jūketsu kyūchūshō no yobō ni kan suru kenkyū 26’, *Kurume igakkai zasshi*, 42, 3 (1979), 290–5.

99
*Chihōbyō to no tatakai*, 1977, 181–2.

100 Komiya Yoshitaka, ‘Miya’irigai (*Oncomelania nosophora*) no seisokuchi to sono satsumetsu no tame no konkuri-toka kōkyo no kanri jōkyō no chōsa’, *Kiseichūgaku zasshi*, 8, 6 (1959), 86–9.

101
*Chihōbyō to no tatakai*, 61.

102
*Chihōbyō no tatakai: Chihōbyō ryūkō shūsoku he no ayumi*, 17.

103 Iida Bunya, Akiyama Takashi, Sasamoto Shōji, and Itō Yasuhiko, *Yamanashi-ken no rekishi* (Tokyo: Deguma shuppansha, 1999), 292–3.

104
*Chihōbyō to no tatakai: Taikensha no shōgen*, 21–3.

105
*Chihōbyō to no tatakai: Taikensha no shōgen*, 22–3.

106 Sasaki Takashi, ‘Nihon jūketsukyūchūbyō bokumetsu taisaku toshite no Miya’irigai seisoku kōkyo no konkuri-toka ni tsuite’, *Kiseichūgaku zasshi*, 7, 5 (1958), 104–05, 111.

107
*Chihōbyō no tatakai: Chihōbyō ryūkō shūsoku he no ayumi*, 16.

108 Iijima Toshihiko, Ito Youichi, and Ishizaki Tatsushi, ‘Epidemiological study on the schistosomiasis japonica among schoolchildren in an endemic area of Yamanashi Prefecture’, *Japanese Journal of Parasitology*, 17, 6 (1968), 71–8, 525–33.

109
*Chihōbyō no tatakai: Chihōbyō ryūkō shūsoku he no ayumi*, 17, 146.

110 Latour, *Aramis*, 212, 280–81.

111 Farley, *Bilharzia*, *Ibid.*, 300.

112 James C. Scott, *Seeing Like a State: How Certain Schemes to Improve the Human Condition Have Failed* (New Haven: Yale University Press, 1998), 4–6.

113 Kitami Chieko, ‘Semento no kawa kirai’, *Yomiuri shinbun*, 12, 17 (1972), 15.

114 Nagata Kiyomi, ‘Chihōbyō wo fusegu semento no kawa’, *Yomiuri shinbun,* 12, 22 (1972), 15.

